# Trends in Methamphetamine Use in the Mainland of China, 2006–2015

**DOI:** 10.3389/fpubh.2022.852837

**Published:** 2022-04-28

**Authors:** Bo Zhang, Xiangyu Yan, Yongjie Li, He Zhu, Zuhong Lu, Zhongwei Jia

**Affiliations:** ^1^School of Public Health, Peking University, Beijing, China; ^2^China Center for Health Development Studies, Peking University, Beijing, China; ^3^State Key Laboratory for Bioelectronics, School of Biological Science and Medical Engineering, Southeast University, Nanjing, China; ^4^Center for Intelligent Public Health, Institute for Artificial Intelligence, Peking University, Beijing, China; ^5^Center for Drug Abuse Control and Prevention, National Institute of Health Data Science, Peking University, Beijing, China

**Keywords:** methamphetamine, drug use, migrants, China, policy

## Abstract

**Background:**

Methamphetamine is the most prevalent drug in the mainland of China, yet few studies on the non-medical use of methamphetamine nationwide have been conducted. This study aimed to examine the trends in the prevalence of non-medical methamphetamine use and to explore the flow pattern of internal migrant methamphetamine users in the mainland of China.

**Methods:**

Data were extracted from the National Dynamic Management and Control Database for Drug Users (NDMCD). Joinpoint regressions were used to examine trends in the prevalence and population size of methamphetamine use.

**Results:**

A total of 1,821,468 methamphetamine use cases registered in NDMCD from 2006 to 2015 were identified. The number of methamphetamine use cases presented an increasing trend in all age subgroups, and among them, the elderly increased the fastest [annual percent change (APC_2006−2015_), 122.9; 95% CI, 113.0–133.3; *p* < 0.001]. The prevalence of methamphetamine use increased from 4.69 per 100,000 population in 2008 to 45.38 per 100,000 population in 2015 (APC_2008−2015_, 36.1; 95% CI, 31.9–40.6; *p* < 0.001). The regions of the Pearl River Delta, Yangtze River Delta, and Beijing-Tianjin-Hebei Urban Agglomeration absorbed over 96% of all internal migrant methamphetamine use cases, and the number of migrant methamphetamine use cases presented increased trends in these three regions.

**Conclusions:**

The increasing trends in methamphetamine use have become a threat to all age groups in China. Substance use prevention programs should focus on internal migrant drug users, especially in economically developed regions.

## Introduction

Methamphetamine use is an increasing public health concern worldwide, particularly in China. According to the United Nations Office on Drugs and Crime, China was one of the countries with the largest quantities of methamphetamine seized globally, and the quantity seized was marked by increases (1). Moreover, the number of methamphetamine users observed nationwide reached up to 1.19 million in 2014 in the mainland of China (2). Methamphetamine use has been found to be associated with long-term damage to human brain cells, and methamphetamine users have a high potential for abuse (3). Therefore, it is imperative to better understand trends and characteristics of methamphetamine use to inform prevention and intervention efforts in China.

There are limited data about national trends and characteristics of methamphetamine use in China. In 2015, China's drug situation reported the number of drug users by drug type for the first time, and among 2.3 million total drug users, 57% (1.3 million) of them used synthetic drugs, which were represented by methamphetamine (4). Previous studies have found that methamphetamine users were more likely to be younger and female than traditional drug users (5, 6). An increasing number of company staffers, entertainers, and students are becoming methamphetamine users (5). Furthermore, methamphetamine use has spread throughout the country, concentrating in southeastern and business-centered cities (5).

Of note, population mobility is the essential cause of the spread of drugs; however, little is known about the impact of migrants on the flow of methamphetamine users. Studies in China and the United States have reported that migrants were at risk of initiating drug use because they often experienced isolation, discrimination, disrupted social networks and drug supply, and they also had barriers to access preventative healthcare services (7). Additionally, the dual epidemics of drug use and infectious diseases have been observed among migrant drug users (8). More studies are needed to better investigate the associations between migration and methamphetamine use in China.

Thus, this study seeks to examine trends in methamphetamine use in the mainland of China and to explore the potential effect of internal migration on the flow of cross-province methamphetamine users. The results will provide management implications for informing drug prevention and control policies in China.

## Methods

### Data Source

The data were drawn from the 2006 to 2015 National Dynamic Management and Control Database for Drug Users (NDMCD). The NDMCD is a database designed to monitor individuals who use drugs for non-medical purposes in the mainland of China. Data in NDMCD are collected by government staff to register observed drug users all around the mainland of China, and the data come from a wide range of sources, such as hospitals, key places (e.g., entertainment venues and stations), and accusations from local people. The NDMCD includes drug users' demographics, drug types, and the characteristics of drug use (e.g., initiation of drug use, location of drug use).

Each drug user in the database was assigned a unique ID encrypted by the SHA-256 algorithm, and no individual persons could be identified directly and indirectly. A drug user may be observed multiple times, and the multiple records could be linked by the unique ID (9).

### Study Variables

Methamphetamine use cases were defined as individuals used methamphetamine for non-medical purposes who were found and registered in NDMCD by the government staff (including policies). To better depict the trends in methamphetamine use over a relatively long period, the number of methamphetamine use case was counted as one if one methamphetamine user was observed more than once within 1 year and was counted as two or more if the same methamphetamine user was observed two or more times across years.

Migrant methamphetamine use cases were identified as methamphetamine use users whose registered province of permanent residence was different from the province where they were observed. In addition, there are three main regions of economic development as well as key inflowing regions of migrants in China: Pearl River Delta (i.e., Guangdong), Yangtze River Delta (i.e., Jiangsu, Zhejiang, and Shanghai), and Beijing-Tianjin-Hebei Urban Agglomeration (i.e., Hebei, Beijing, and Tianjin) (Supplementary Figure S1).

Demographic information included age, sex (male and female), education (primary school or below, junior high school, and high school or above), marital status (divorced or widowed, married, and unmarried), occupation (employee, worker, farmer, freelancer, self-employed, and unemployed), and drug use sites (hotels, home, car, bathing room, entertainment venues, and others).

### Statistical Analysis

First, the annual number of methamphetamine use cases was accumulated cases every calendar year. The rate of methamphetamine use cases was calculated by the annual number of methamphetamine use cases (numerator) divided by the population (denominator) within the same calendar year. The population numbers were obtained from the statistical yearbook (10). The trends in the number and rate of methamphetamine use cases were described, stratified by age group, sex, and migrant status. Next, joinpoint regressions were used to examine trends in the annual number and rate of methamphetamine use cases during the study period. The annual percent change (APC, %) was calculated by joinpoint regression to show the significance of the trend over time. The joinpoint program starts with the minimum number of joinpoints (e.g., 0 joinpoints, which is a straight line) and tests whether more joinpoints must be added to the model, with the goal of detecting a statistically significant change in the trend (11, 12).

A two-sided *p* value of 0.05 or less was regarded as significant. Descriptive analyses were performed in SPSS 22.0, joinpoint regressions were conducted in Joinpoint Regression Program 4.6.0, and maps were generated in ECHARTS 4.0.

## Results

### Characteristics of the Study Sample

Overall, 1,821,468 methamphetamine use cases between 2006 and 2015 were included in the analysis, and among them, 80.9% were male, 81.8% had an education level of junior high school or below, 52.2% were unmarried, 52.8% were unemployed, 42.2% used drugs at home, and the median age was 28 years old [inter-quartile range (IQR), 23–35 years old]. In addition, the proportion of male methamphetamine use cases increased from 75.2% in 2006 to 84.1% in 2015 (Table 1).

**Table 1:** Sociodemographic characteristics of methamphetamine use cases: 2006–2015 in the mainland of China.

**Characteristics**	**Total**	**2006**	**2009**	**2012**	**2015**
**Total**	**1,821,468**	**13,455**	**104,465**	**247,015**	**622,038**
Sex (%)					
Female	19.1	24.8	24.8	19.7	15.9
Male	80.9	75.2	75.2	80.3	84.1
Age (IQR^a^)	28 (23–35)	28 (23–35)	27 (22–35)	28 (23–35)	30 (25–37)
Education (%)					
High school or above	10.9	5.9	9.4	11.8	11.5
Junior high school	65.2	30.9	53.3	71.4	68.2
Primary school or below	16.6	7.2	10.5	16.7	18.2
Missing	7.3	56.1	26.7	0.1	2.1
Marital status (%)					
Married	35.9	22.4	32.0	36.6	39.6
Unmarried	52.2	15.3	37.8	57.7	54.0
Divorced/Widows	5.4	4.0	3.7	5.8	6.4
Missing	6.5	58.3	26.5	0.0	0.0
Occupation (%)					
Employee	1.0	0.6	0.6	1.2	1.1
Worker	3.0	0.9	1.3	2.8	3.6
Farmer	8.9	4.6	5.0	9.2	9.5
Freelancer	2.3	1.0	2.1	2.2	2.5
Self-employed	2.8	1.6	1.8	3.3	3.0
Unemployed	52.8	23.8	44.3	59.4	54.6
Others	22.7	9.2	18.4	22.0	25.7
Missing	6.5	58.4	26.5	0.0	0.0
Drug use sites (%)					
Hotels	29.5	28.8	42.4	34.8	24.3
Home	42.2	21.9	34.0	40.1	45.4
Car	1.6	0.0	0.0	0.0	2.6
Bathing room	0.5	3.6	2.8	0.3	0.1
Entertainment venues	5.2	12.8	8.1	5.6	4.1
Others	19.9	10.1	11.5	18.7	23.3
Missing	1.1	22.7	1.2	0.4	0.2

a *IQR, inter-quartile range*.

### Trends in Methamphetamine Use Cases

**Figure 1:**
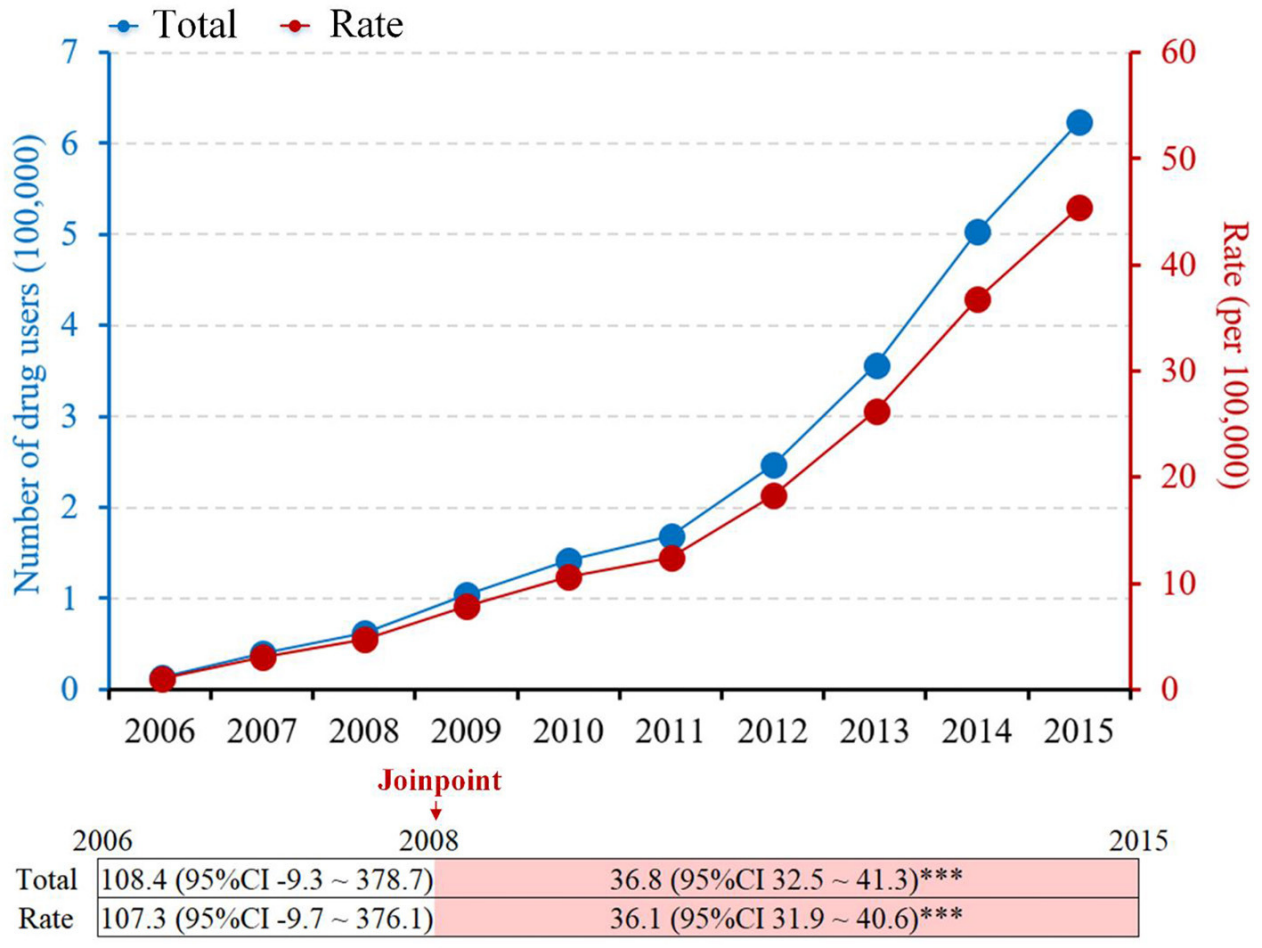
Trends in the annual number and rate of methamphetamine use cases: 2006–2015 in the mainland of China (***indicated *p* < 0.001).

Both the annual number and rate of methamphetamine use cases showed significant increasing trends from 2008 to 2015 in the mainland of China. The annual number of methamphetamine use cases was stable from 2006 to 2008 (APC, 108.4; 95% CI −9.3 to 378.7; *p* > 0.05), then increased from 62,251 in 2008 to 623,765 in 2015 (APC, 36.8; 95% CI, 32.5–41.3; *p* < 0.001), and the rate was also stable before 2008 but increased from 4.69 per 100,000 population in 2008 to 45.38 per 100,000 population in 2015 (APC, 36.1; 95% CI, 31.9–40.6; *p* < 0.001) (Figure 1).

### Demographic Trends in Methamphetamine Use Cases

**Figure 2:**
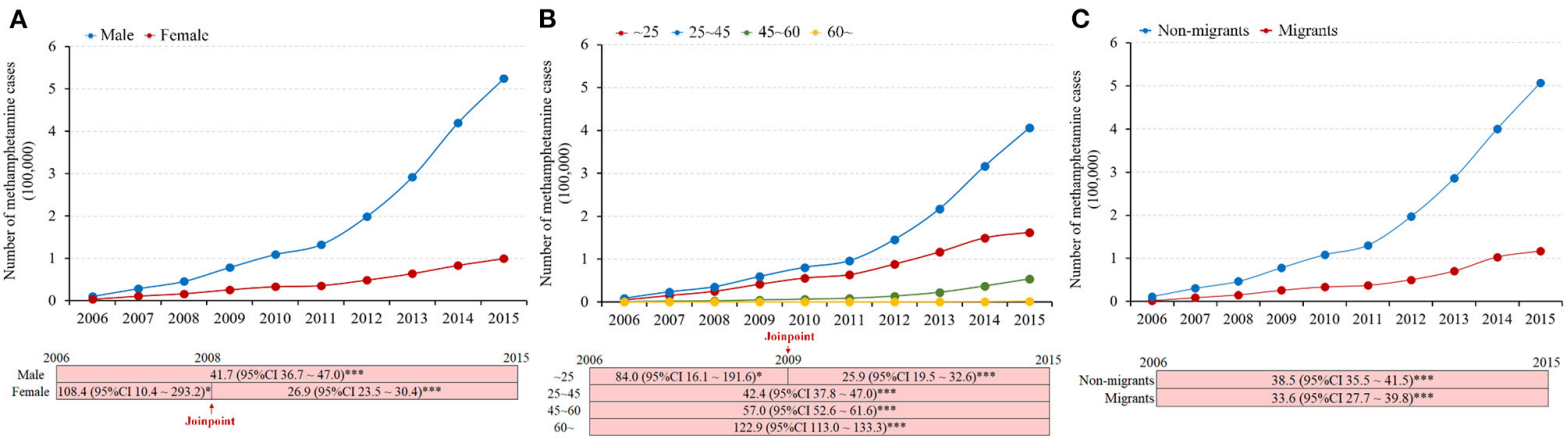
Trends in the annual number of methamphetamine use cases, stratified by sex **(A)**, age groups **(B)**, and migrant status **(C)**, 2006–2015 in the mainland of China (*indicated *p* < 0.05; ***indicated *p* < 0.001).

The annual number and rate of both male and female methamphetamine use cases presented upward trends (Figure 2A; Supplementary Figure S2). The number of male methamphetamine use cases [from 10,127 in 2006 to 524,458 in 2015 (APC, 41.7; 95% CI, 36.7–47.0; *p* < 0.001)] increased faster than that of female cases [from 3,335 in 2006 to 99,307 in 2015 (APC_2006−2008_, 108.4; 95% CI, 10.4–293.2; *p* < 0.05; APC_2008−2015_, 26.9; 95% CI, 23.5–30.4; *p* < 0.001)] (Figure 2A).

The annual number and rate of methamphetamine use cases presented increasing trends in all age subgroups (Figure 2B; Supplementary Figure S2). The number of elderly people aged >60 years old increased from 1 in 2006 to 1,544 in 2015 (APC, 122.9; 95% CI, 113.0–133.3; *p* < 0.001), which was the fastest among the age subgroups. Other age subgroups also had disproportional increases (45–60 years old: APC, 57.0; 95% CI, 52.6–61.6; *p* < 0.001; 25–45 years old: APC, 42.4; 95% CI, 37.8–47.0; *p* < 0.001; <25 years old: APC_2006−2009_, 84.0; 95% CI, 16.1–191.6; *p* < 0.05 and APC_2009−2015_, 25.9; 95% CI, 19.5–32.6; *p* < 0.001) (Figure 2B).

The annual number of cross-province migrant methamphetamine use cases increased from 202 in 2006 to 116,589 in 2015 (APC, 33.6; 95% CI, 27.7–39.8; *p* < 0.001), and the annual number of non-migrant methamphetamine use cases increased from 11,442 in 2006 to 507,176 in 2015 (APC, 38.5; 95% CI, 35.5–41.5; *p* < 0.001) (Figure 2C).

### Trends in Regional Mobility of Methamphetamine Use Cases

**Figure 3:**
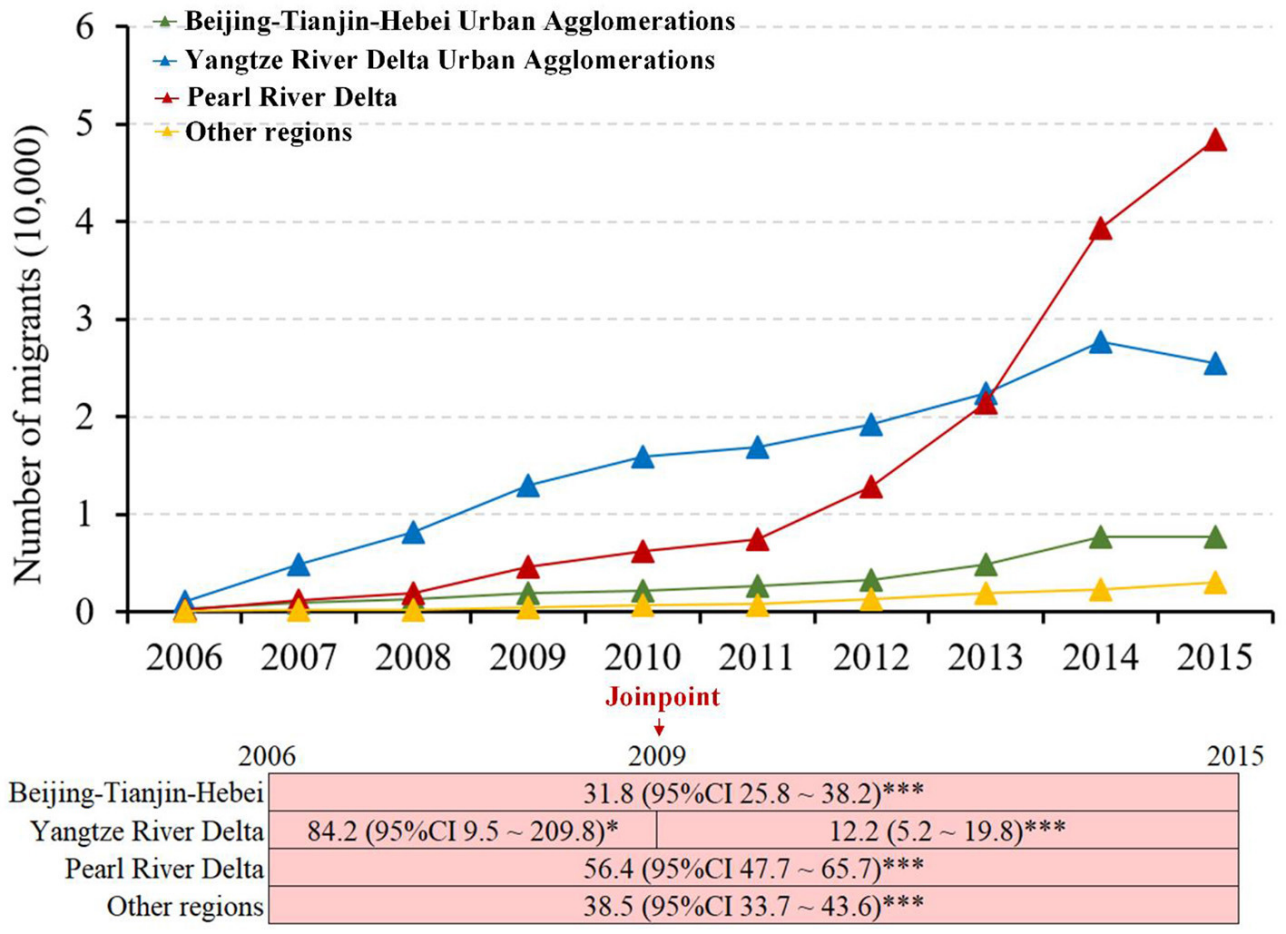
Trends in the annual number of migrant methamphetamine use cases, stratified by destination geographical areas: 2006–2015 in the mainland of China (*indicated *p* < 0.05; ***indicated *p* < 0.001).

Before 2010, methamphetamine use was more prevalent in a few provinces along the southeastern coast. In 2015, methamphetamine use cases were mainly distributed in certain regions in China (Supplementary Figure S3). The Pearl River Delta, Yangtze River Delta, and Beijing-Tianjin-Hebei Urban Agglomeration absorbed over 95% of all internal migrant methamphetamine use cases (Supplementary Table S1). The annual number of migrant methamphetamine use cases to the Pearl River Delta increased the fastest from 227 in 2006 to 48,420 in 2015 (APC, 56.4; 95% CI, 47.7–65.7; *p* < 0.001), followed by the Beijing-Tianjin-Hebei Urban Agglomeration from 341 in 2006 to 7,680 in 2015 (APC, 31.8; 95% CI, 25.8–38.2; *p* < 0.001), and the Yangtze River Delta from 1,029 in 2006 to 25,449 in 2015 (APC_2006−2009_, 84.2; 95% CI, 9.5–209.8; *p* < 0.05; APC_2009−2015_, 12.2; 95% CI, 5.2–19.8; *p* < 0.001) (Figure 3).

**Figure 4:**
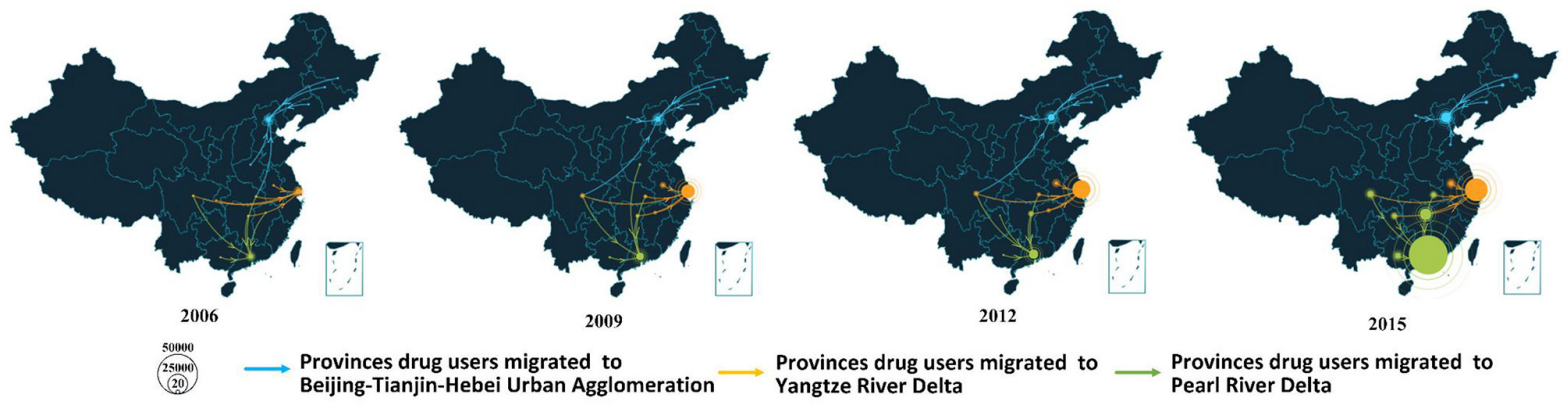
The flow of methamphetamine use cases migrating to regions of Beijing-Tianjin-Hebei Urban Agglomeration, Yangtze River Delta, and Pearl River Delta (the arrow indicated the direction of migration among methamphetamine cases. The radius of the destination circle indicated the number of methamphetamine cases migrated into this region. The radius of the source circle indicated the number of methamphetamine cases migrated out this region).

The proportion of migrants observed in the Yangtze River Delta and Beijing-Tianjin-Hebei Urban Agglomeration among all migrant methamphetamine use cases showed annual decreasing trends, while the Pearl River Delta presented an increasing trend (Figure 3). The annual number of migrant methamphetamine use cases to Pearl River Delta exceeded that of the Yangtze River Delta in 2014 (Figure 3). The main source regions of migrant methamphetamine users in the three regions presented very different characteristics. In detail, the permanent residence of migrant methamphetamine users flowing to the Beijing-Tianjin-Hebei Urban Agglomeration region was mainly located in northeast China, and migrant methamphetamine users in Pearl River Delta and Yangtze River Delta regions mainly came from southwest and central China (Figure 4).

## Discussion

Methamphetamine has become the most common drug used in China, and it accounts for more than half of total drug use (4). In this study, we found that both the annual number and rate of methamphetamine use cases showed rising trends in the total sample and among female and all age subgroups. Although the annual number of elderly methamphetamine users was not as high as that of younger users, the rate of methamphetamine use increased the fastest. The economically developed regions of the Pearl River Delta, Yangtze River Delta, and Beijing-Tianjin-Hebei Urban Agglomeration are areas with high population density and a developed economy in China. Internal migrant drug users mainly flowing to the three regions have brought challenges to local society management, which deserves attention.

The prevalence of methamphetamine use maintained a rapid growth, and it exceeded the prevalence of traditional opioid use in 2014 and became a new drug issue in China (2). It was reported that the reversed trend from opioids to methamphetamine occurred from east to the west China, and methamphetamine became the main drug used in each province (13). Before the economic reform and opening-up, there was no circulation of methamphetamine in the mainland of China (14, 15). Until the 1990s, methamphetamine flowed to the Pearl River Delta from abroad, which was the most open and developed area in China at that time (16). Guangdong province in the agglomeration is famous for drug manufacturing, especially for methamphetamine manufacturing. Later, methamphetamine from abroad and the southeast coast of China (e.g., Guangdong) flowed into the inland regions and gradually expanded, which contributed to the changing trend in methamphetamine and heroin use (5).

Monitoring drug use is difficult due to the hidden and complex nature of drug use behaviors in China. The wastewater-based epidemiology (WBE) was introduced in 2001 and first implemented in 2005 (17). The first WBE study in the mainland of China was carried out in 2012. Wastewater samples collected in four megacities suggested that methamphetamine use was particularly noteworthy, and they indicated a geographical trend that methamphetamine use was more prevalent in southern China than in northern China (18). A nationwide investigation of wastewater samples were collected from 36 sewage treatment plants in 18 major cities, and the results indicate that methamphetamine use have increased substantially in Beijing, Shanghai, and Shenzhen since 2012 (19). In our study, we found that the prevalence of methamphetamine use has been increasing since 2008, especially in the east, and it spread from east to west, which were consistent with these WBE studies. Moreover, data from NDMCD also further described drug users in terms of sociodemographic characteristics (e.g., age, sex) and drug use behaviors (e.g., injection, inhalation), which beyond the information provided by WBE.

All age groups showed an increasing trends in methamphetamine use, especially for young and elderly users (15). Previous studies found that seeking entertainment and recreational use enabled methamphetamine to become more popular among young drug users (20, 21). Also, we found that the annual number of methamphetamine use cases in elderly subgroups presented significantly faster increasing trends. The United Nations Office on Drugs and Crime reported that the prevalence of drug use among the elderly has been on the rise and has grown faster than that among younger people in the past decade in some countries (22, 23). For example, in the United States, the number of lifetime drug users aged over 50 years was 12 times as many as that in 1996 (22, 23). Studies have suggested that the increasing trend in methamphetamine use among the elderly could be attributed to external and internal factors. The external factor was the “baby boom,” which referred to the phenomenon of a 300% increase in the birth rate during 1952–1959 in China. In the 1980s, as the midst of the reform and opening-up, China was invaded by the methamphetamine epidemic, and during this period, the baby boomers entered their youth (21–28 years old) and became the largest victim population (24). Internal factors were mainly related to the characteristics of the elderly population, including iatrogenic drug use, psychological problems, and lack of awareness about drugs (25).

The primary prevention of methamphetamine use for the elderly population should focus on reducing and eliminating the risk factors, guiding elderly people to maintain a good lifestyle and healthy mental state to reduce the incidence of methamphetamine use. First, official departments should strengthen supervision of the whole process of methamphetamine production, circulation and sale, to eliminate underground drug trading networks [43, 44]. Second, it should promote the publicity and education of the addiction mechanism of methamphetamine in communities (26). The secondary prevention is to achieve early detection, intervention, and treatment to reduce the harm of methamphetamine use. Secondary prevention is mainly targeted at the elderly congregated communities and high-risk populations with a history of methamphetamine use. Family doctors should be aware of identifying and judging the risk of methamphetamine use according to the physical and mental state of the elderly (27). For occasional use among elderly methamphetamine users, early intervention combined with behavioral intervention and clinical medication can prevent addiction and serious health outcomes (28, 29). Tertiary prevention aims to reduce the harmful effects on society and public health triggered by methamphetamine addiction and relapse. Besides providing the treatment for methamphetamine users in medical institutions, it is necessary to train their families to strengthen the accompany and supervision to encourage them to regain confidence and seek detoxification timely. It is necessary for the government to monitor elderly methamphetamine users and establish social support networks (e.g., communities, families and hospitals) and more rehabilitation institutions and self-help organizations (28–30).

Research in the field of methamphetamine use among elderly people is still insufficient. First, evidences of the epidemic trend and risk factors for methamphetamine use among the elderly population is scarce. Second, few studies quantitatively evaluated the effect of baby boomer on the methamphetamine epidemic among the elderly population. Third, research contents, including the dose, duration and health effects of methamphetamine use among the elderly population, should be further improved.

To our knowledge, this study was the first to focus on the issues caused by the internal migration of methamphetamine users in China. Methamphetamine users had higher mobility, and the proportion of internal migrant methamphetamine users among total methamphetamine users was ~20–25%. Over 95% of migrant drug users migrated into three regions: Pearl River Delta, Yangtze River Delta, and Beijing-Tianjin-Hebei Urban Agglomeration, and migrant methamphetamine users in the Pearl River Delta increased the fastest.

The increasing trend in methamphetamine use in the Pearl River Delta was more likely to be associated with three factors: manufacturing, transportation, and migrant population. First, rural areas in the Pearl River Delta were China's most active methamphetamine manufacturing regions. High drug profits have led some underdeveloped rural areas astray and fostered government corruption (31). Second, about 90% of people and goods enter China through the Pearl River Delta, which may lead to a drug trafficking market. Prior to 2014, the region supplied one-third of China's methamphetamine (31). Last, the number of observed methamphetamine users increased sharply in Pearl River Delta, which might increase the risk of methamphetamine use among internal migrants. Therefore, there is a need for a regular application of the law by professional law enforcers supported by government and greater investment in human development to expand opportunities for vulnerable townships and villages in the Pearl River Delta.

In addition, due to cultural differences in arrival communities, migrants tend to be subjected to discrimination and marginalization, which may make methamphetamine users feel isolation and disrupt social ties. Therefore, society should be more tolerant of migrants and migrant drug users and optimize settlement mechanisms. It is essential to strengthen the training of drug users in a variety of skills to reduce poverty and unemployment and control entertainment places where methamphetamine is highly spread to reduce incarceration rates. It is important to increase the coverage of affordable housing and reduce homelessness. Additionally, establishing a cross-regional health insurance system to ensure drug users' access to healthcare access or harm reduction services could also be an improvement of intervention and managements of migrant drug users.

### Limitation

There were several limitations in this study. First, we only used the 2006–2015 data mainly due to the nature and availability of the database. Although the data are becoming outdated, it is of great public health significance to report relatively early data on illicit drug use, which can fill in the data and research gaps in China. Second, due to the nature of NDMCD data, our estimates of the number of methamphetamine users were underestimated and conservative. However, there were no better data sources of drug use in China to describe the trends in specific drug use. Third, due to the use of the accumulated data, we cannot explore the factors associated with methamphetamine use. Fourth, migrants may have several internal migrations within 1 year, and we cannot catch up every flow, which may affect the understanding of the flow of migrants. However, to our knowledge, this is the first study on the epidemic of methamphetamine use based on the national registry database in the mainland of China. Such a work of evaluating a real database can help the control and prevention of drug use issues. Last, the COVID-19 pandemic has had a significant impact on the drug market, and future studies are needed to focus on methamphetamine use during the COVID-19 period.

## Conclusions

The epidemic of methamphetamine was the most important issue of substance use in the mainland of China. Our findings showed an increasing trend in methamphetamine use in China from 2006 to 2015, especially among the elderly, and tertiary prevention should be strengthened. Substance use prevention programs should focus on internal migrant drug users, especially in economically developed regions.

## Data Availability Statement

The data analyzed in this study is subject to the following licenses/restrictions: this study is a secondary data analysis. The data we have was anonymized by the government to protect the privacy of persons who use drugs. Government authorization is needed to access the dataset. The datasets used and analyzed during the current study are available from the corresponding author on reasonable request. Requests to access these datasets should be directed to BZ, zhangbo0136@pku.edu.cn.

## Author Contributions

ZJ and ZL designed the study. BZ and XY cleaned the data. BZ, XY, and YL analyzed the data. ZJ, ZL, BZ, and HZ explained the results. ZJ and BZ wrote the initial draft of the manuscript. ZJ and HZ revised the report from preliminary draft to submission. All authors read and approved the final manuscript.

## Funding

This study was supported by the National Natural Science Foundation of China (Grant Numbers 72104008, 91546203, and 91846302), the Chinese Ministry of Public Security (0716-1541GA590508), and the Ministry of Science and Technology of the People's Republic of China.

## Author Disclaimer

The opinions expressed herein show the collective views of the coauthors and do not necessarily represent the official position of funding.

## Conflict of Interest

The authors declare that the research was conducted in the absence of any commercial or financial relationships that could be construed as a potential conflict of interest.

## Publisher's Note

All claims expressed in this article are solely those of the authors and do not necessarily represent those of their affiliated organizations, or those of the publisher, the editors and the reviewers. Any product that may be evaluated in this article, or claim that may be made by its manufacturer, is not guaranteed or endorsed by the publisher.
