# Supplementary material for: Trends in Methamphetamine Use in the Mainland of China, 2006–2015
Source: Front Public Health. 2022 Apr 28;10:852837. doi: 10.3389/fpubh.2022.852837 (PMC9096246; doi:10.3389/fpubh.2022.852837)
Supplement: Supplementary file 1 [file Image_1.pdf]

Appendix

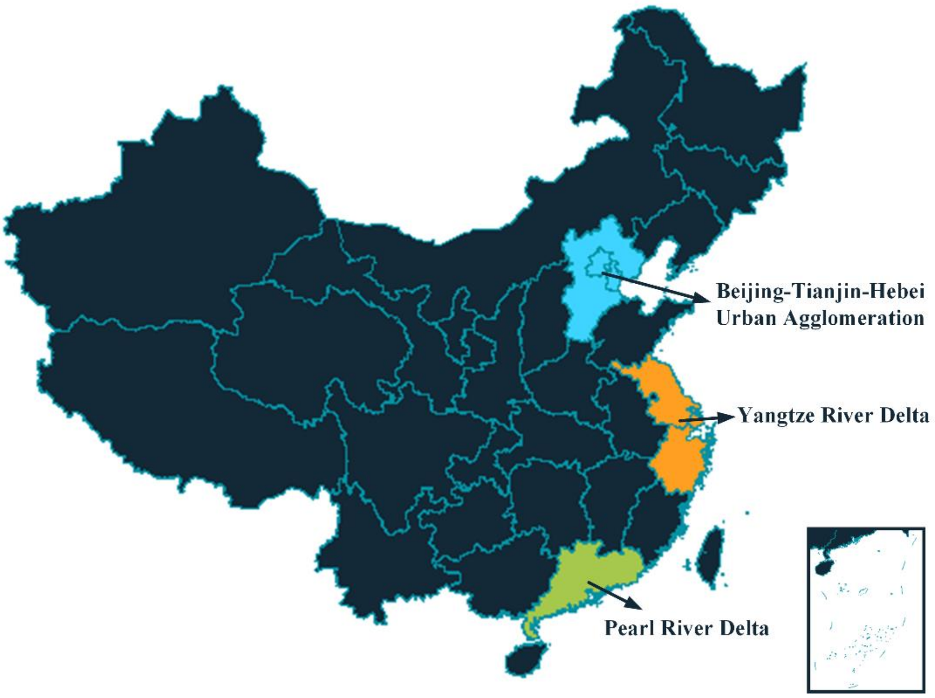

Figure S1. Three main regions of the economic development in the mainland of China

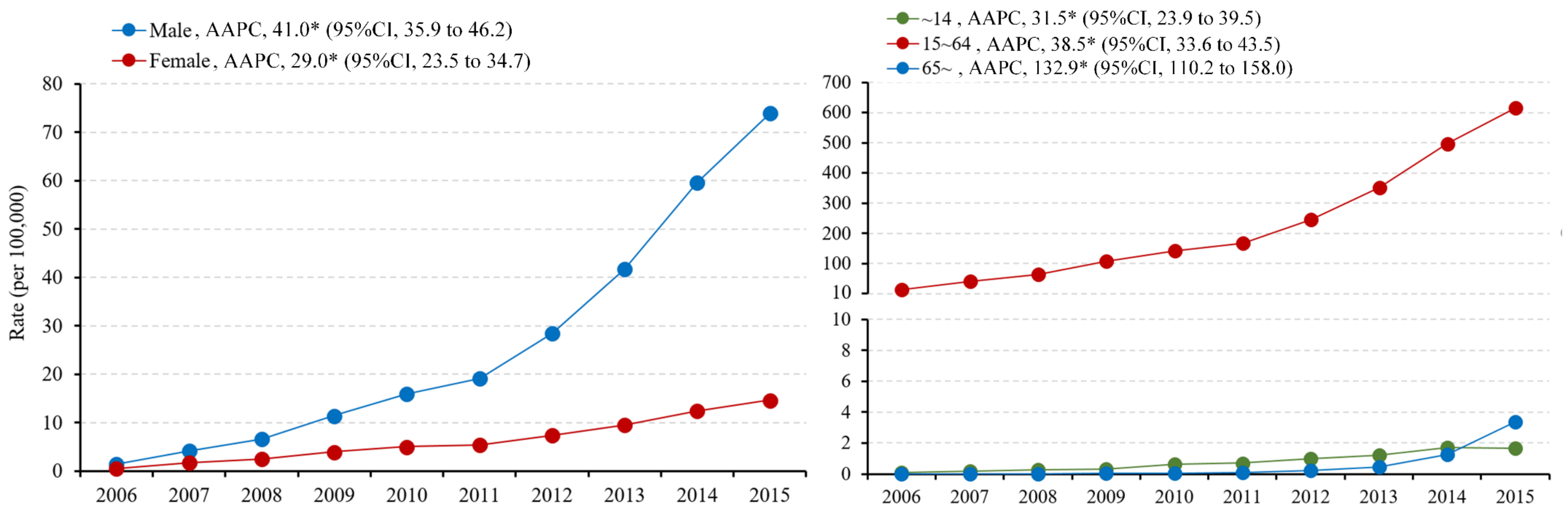

Figure S2. Trends in prevalence of methamphetamine use stratified by sex and age

(* indicated p<0.05)

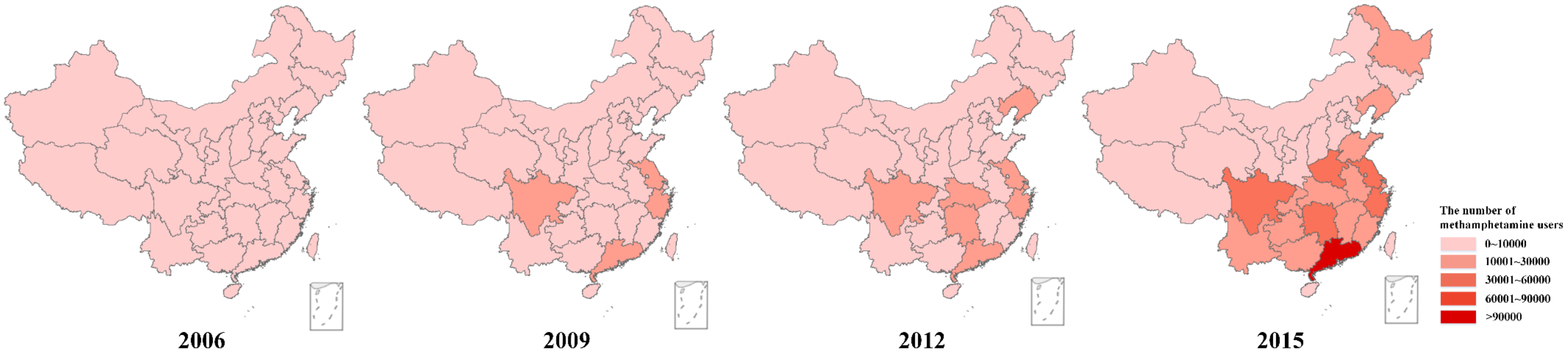

Figure S3. Trends of the number of methamphetamine cases in regions

Table S1. The proportion of migrants among total migrant methamphetamine users (%)

	2006	2007	2008	2009	2010	2011	2012	2013	2014	2015
Pearl River Delta	13.5	16.1	16.6	23.0	24.7	26.9	35.0	42.4	51.0	57.2
Yangtze River Delta	61.1	67.2	70.1	64.7	63.7	60.5	52.4	44.3	36.0	30.1
Beijing-Tianjin-Hebei Urban Agglomeration	20.2	13.2	10.9	9.7	8.9	9.8	9.1	9.6	10.0	9.1
Other regions	5.2	3.5	2.4	2.6	2.7	2.8	3.5	3.7	3.0	3.7
